# Distribution and Clinical Impact of *Helicobacter pylori* Virulence Factors in Epstein–Barr-Virus-Associated Gastric Cancer

**DOI:** 10.3390/antibiotics14060580

**Published:** 2025-06-05

**Authors:** Jin Hee Noh, Ji Yong Ahn, Hee Kyong Na, Jeong Hoon Lee, Kee Wook Jung, Do Hoon Kim, Kee Don Choi, Ho June Song, Gin Hyug Lee, Hwoon-Yong Jung

**Affiliations:** 1 Department of Internal Medicine, Hallym University Sacred Heart Hospital, Hallym University College of Medicine, Anyang 14068, Republic of Korea; mdjh1432@gmail.com; 2 Department of Gastroenterology, Asan Medical Center, University of Ulsan College of Medicine, Seoul 05505, Republic of Korea; hkna77@naver.com (H.K.N.); jhlee.gi@amc.seoul.kr (J.H.L.); jung.keewook30@gmail.com (K.W.J.); dohoon.md@gmail.com (D.H.K.); keedonchoi@gmail.com (K.D.C.); hjsong@amc.seoul.kr (H.J.S.); jhlee409@amc.seoul.kr (G.H.L.); hwoonymd@gmail.com (H.-Y.J.)

**Keywords:** Epstein–Barr virus, *Helicobacter pylori*, stomach neoplasms, virulence factors

## Abstract

**Background**: *Helicobacter pylori* (HP) and Epstein–Barr virus (EBV) coinfection lead to chronic inflammation and contribute to the development of gastric cancer. However, studies examining the association between HP virulence factors and EBV infection in gastric cancer are limited. This study investigated the polymorphisms of HP virulence factors associated with EBV infection and their effects on clinical outcomes in EBV-associated gastric cancer (EBVaGC). **Methods**: A total of 96 HP isolates from 54 patients with gastric cancer were divided and analyzed based on EBV coinfection status. Polymerase chain reaction amplifications of virulence factors were conducted using DNA extracts from HP isolates cultured from gastric mucosal specimens. **Results**: EBV infection was significantly associated with gastric carcinoma with lymphoid stroma morphology and a proximal location in the stomach. Most HP strains from patients with gastric cancer were positive for *cagA* (100.0%), *vacA* (100.0%), and *iceA*1 (87.5%). Among HP isolates with EBV coinfection, the prevalence of *iceA*2 (21.7% vs. 0.0%, *p* < 0.001) and *ureA* (21.7% vs. 4.0%, *p* = 0.009) was significantly more frequent, and that of *iceA*1 (78.3% vs. 96.0%, *p* = 0.009) and *vacA* s1a (4.3% vs. 22.0%, *p* = 0.012) was less frequent than those of EBV– colonies. Multivariate analysis indicated that *ureA* (odds ratio, 6.148; 95% confidence interval [CI], 1.221 to 30.958; *p* = 0.028) was associated with EBVaGC. No significant difference in clinical outcomes was observed based on the presence of *ureA* expression in EBVaGC. **Conclusions**: In gastric cancer, regardless of EBV infection, most HP strains were highly virulent, testing positive for *cagA*, *vacA*, and *iceA*1. Although *ureA* was significantly associated with EBV infection, it did not influence the clinical outcomes of EBVaGC.

## 1. Introduction

Gastric cancer remains a significant global health concern, particularly in East Asia, where incidence and mortality rates are notably high. According to the Global Cancer Statistics (GLOBOCAN 2022), it ranks as the third-most commonly diagnosed cancer and the fourth leading cause of cancer mortality in South Korea [1]. While multiple genetic and environmental risk factors contribute to gastric carcinogenesis, infectious agents have emerged as crucial contributors [2,3].

*Helicobacter pylori* (HP), classified as a class I carcinogen by the World Health Organization in 1994, is recognized as a primary causative agent in the development of gastric cancer [4]. HP infection initiates a pathological cascade that progresses from chronic gastritis to atrophic gastritis, intestinal metaplasia, and dysplasia, ultimately leading to intestinal-type gastric cancer [5]. The pathogenicity of HP is influenced by various virulence factors, including *cagA*, *vacA*, *iceA*, *oipA*, and *dupA*, which show significant associations with gastric cancer development [6,7].

Recent studies have also identified Epstein–Barr virus (EBV) infection as a risk factor in gastric carcinogenesis [8]. Notably, coinfection with EBV and HP has been reported to significantly increase the risk of developing gastric cancer by synergistically inducing severe inflammatory responses in the gastric tissue [9]. Despite several studies examining the role of HP virulence factors in gastric carcinogenesis, limited research has addressed the relationship between these factors and EBV infection in gastric cancer.

Therefore, we aimed to analyze the genotypes of major HP virulence factors in patients with gastric cancer with EBV and HP coinfection, as well as the effects of these virulence factors on the clinical outcomes of EBV-associated gastric cancer (EBVaGC).

## 2. Results

### 2.1. Clinicopathologic Characteristics According to EBV Infection Status

Table 1 outlines the clinical characteristics of patients stratified by EBV status (EBV+ [n = 28], EBV– [n = 26]). The median age of all patients was 62 (IQR, 55–68) years, with males comprising 83.3% of all patients. HP eradication success rates were not significantly different between the EBV+ and EBV–groups (88.2% vs. 93.8%, *p* = 1.000). Tumors in the EBV+ group were more frequently located in the upper stomach compared to the EBV– group (28.6% vs. 7.7%, *p* = 0.034). Histologically, the GCLS type was significantly more prevalent in the EBV+ group (53.6% vs. 0.0%, *p* < 0.001). No significant differences were observed between the two groups in terms of recurrence-free survival (31.5 vs. 31.0 months, *p* = 0.075) or overall survival (32.0 vs. 32.5 months, *p* = 0.113).

### 2.2. Positivity of Virulence Factors According to EBV Infection Status

**Figure 1:**
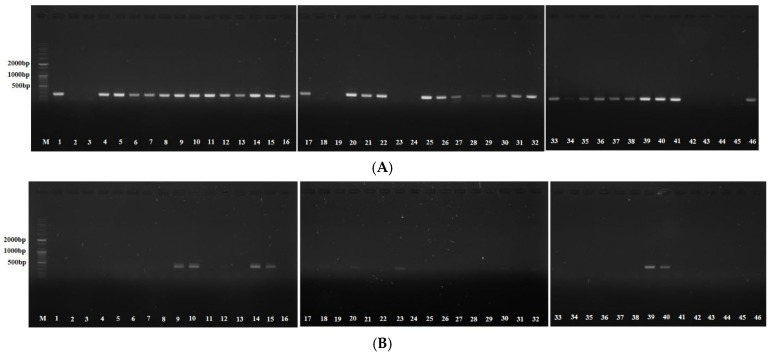
Agarose gel electrophoresis for polymerase chain reaction products of *Helicobacter pylori* virulence factors in Epstein–Barr-virus-positive colonies. (**A**) *iceA*1 (247 bp) (−): 2/3/18/19/23/24/42/43/44/45. (**B**) *ureA* (411 bp) (+): 9/10/14/15/18/20/23/30/39/40. DNA size marker: 2000 bp, 1000 bp, 500 bp.

All HP isolates from patients with gastric cancer tested positive for *cagA* (100.0%) and *vacA* (100.0%), with *iceA*1 detected in 87.5% of isolates. Among HP isolates from patients with EBV coinfection, *iceA*2 (21.7% vs. 0.0%, *p* < 0.001) and *ureA* (21.7% vs. 4.0%, *p* = 0.009) were significantly more frequent, and *iceA*1 (78.3% vs. 96.0%, *p* = 0.009), *JHP917* (0.0% vs. 18.0, *p* = 0.003), and *vacA* s1a (4.3% vs. 22.0%, *p* = 0.012) were less frequent compared to the EBV– group (Table 2). Multivariate analysis indicated that *ureA* (OR, 6.148; 95% CI, 1.221 to 30.958; *p* = 0.028) was significantly associated with EBVaGC (Table 3), whereas *iceA*1 showed an inverse association (OR, 0.163; 95% CI, 0.032 to 0.819; *p* = 0.028). Figure 1 presents the agarose gel electrophoresis results for *iceA*1 and *ureA*.

### 2.3. Clinical Outcomes According to Virulence Factors in EBV-Associated Gastric Cancer

We analyzed clinical outcomes based on the presence of *ureA* in patients with both EBV and HP coinfection (n = 46) (Table 4). Despite multivariate analysis confirming a significant association between *ureA* and EBVaGC, no significant differences in clinical outcomes were observed based on *ureA* expression. Supplemental Tables S1 and S2 summarize the comparisons of clinical outcomes based on the presence of *iceA*1 and *iceA*2. Similarly, no significant differences in clinical outcomes were detected with respect to the presence of *iceA*1 and *iceA*2.

## 3. Discussion

In this study, regardless of EBV infection, HP isolates from patients with gastric cancer exhibited highly virulent factors, including *cagA*, *vacA*, and *iceA*1. In cases of EBV and HP coinfection, the HP isolates displayed *iceA*2 and *ureA* virulence factors more frequently than those with HP infection alone, with *ureA* showing a significant association with EBVaGC. However, the presence of *ureA* did not lead to significant differences in the clinical outcomes of EBVaGC.

HP has been linked to various malignant conditions, including gastric carcinoma and mucosa-associated lymphoid tissue lymphoma of the stomach. Concurrently, EBV can induce oncogenic transformation in infected cells by activating multiple signaling pathways [10]. The coinfection of these two pathogens may exert synergistic effects, enhancing inflammatory responses in gastric tissues and increasing the risk of gastric cancer development [11,12,13]. We have reported previously that EBV and HP coinfection does not significantly influence clinical outcomes in patients with EBVaGC [14]. However, there is a lack of studies investigating which HP virulence factors are prominent in EBVaGC and their impact on clinical outcomes.

Consistent with prior studies, our study found that EBV+ gastric cancers were predominantly located in the proximal region of the stomach and exhibited GCLS histology more frequently than the EBV– group [14,15]. A notable finding from our study is that HP isolates from patients with gastric cancer, irrespective of EBV infection status, were predominantly highly virulent strains positive for *cagA* (100%), *vacA* (100%), and *iceA*1 (87.5%). This high prevalence of virulent strains supports the well-established association between HP virulence factors and increased gastric cancer risk [7,16,17,18]. Among these factors, *cagA* has been reported to enhance EBV-driven epigenetic modifications by increasing DNA methyltransferase expression and promoting gastric carcinogenesis through hypermethylation and silencing of tumor suppressor genes related to cell cycle regulation, apoptosis, and DNA repair [16].

Our study revealed significant differences in the distribution of virulence factors between the EBV+ and EBV– groups. EBV-coinfected HP isolates exhibited a significantly higher prevalence of *iceA*2 and *ureA* while demonstrating lower frequencies of *iceA*1, *JHP917*, and *vacA* s1a. These findings suggest that specific HP virulence profiles may interact differently with EBV infection, potentially contributing to distinct carcinogenic pathways. Furthermore, multivariate analysis identified *ureA* (OR 6.148) as significantly associated with EBV+ gastric cancer. The urease enzyme encoded by the *ure* gene cluster, including *ureA*, is essential for HP colonization by neutralizing gastric acid, thus creating a favorable microenvironment for bacterial survival [19,20,21,22]. Further studies are required to confirm whether this enhanced urease activity facilitates EBV infection or reactivation in the gastric mucosa. Despite the significant association between *ureA* and EBV+ gastric cancer, our analysis found no differences in clinical outcomes, including recurrence-free survival and overall survival, based on *ureA* expression status. This indicates that although these virulence factors may influence susceptibility to EBV and HP coinfection and the development of gastric cancer, they may not significantly affect disease progression or prognosis. Recent in vitro studies have demonstrated that EBV infection enhances phosphorylation-dependent CagA activity and amplifies the oncogenic potential of HP [23,24]. These findings suggest that EBV and HP coinfection may involve complex molecular interactions beyond simple co-colonization, indicating that the differential virulence factor profiles observed in this study are meaningful and warrant further investigation.

This study has certain limitations. First, the retrospective design and relatively small sample size may limit the generalizability of our findings and reduce statistical power, particularly for subgroup analyses. Second, the enrolled patients predominantly consisted of early gastric cancer cases (94.4% in the EBV+ and 92.3% in the EBV– groups), which may not represent the full spectrum of gastric cancer progression. Third, because HP isolates were analyzed from patients with diagnosed gastric cancer, it was not possible to evaluate the role of virulence factors in the early stages of carcinogenesis or the temporal relationship with EBV infection. It remains unclear whether specific HP virulence profiles predispose individuals to EBV infection or whether EBV infection exerts selective pressure favoring certain HP strains.

## 4. Materials and Methods

### 4.1. Study Population

We analyzed HP isolates obtained from patients with gastric cancer who visited Asan Medical Center between January 2018 and February 2021. The inclusion criteria were as follows: (1) age ≥ 19 years; (2) histologically confirmed gastric cancer; (3) HP infection confirmed by culture from endoscopic biopsy specimens; and (4) for the EBV+ group, EBV positivity confirmed by in situ hybridization in tumoral gastric mucosa. The exclusion criteria were as follows: (1) age < 19 years; and (2) cases without culture-confirmed HP infection. A total of 96 HP isolates obtained from 54 patients with gastric cancer were enrolled and analyzed. They were divided into two groups: patients with HP and EBV coinfection (28 patients, 46 isolates) and those with HP infection alone (26 patients, 50 isolates). Clinicopathological data for each patient were retrospectively collected from medical records. The study protocol was approved by the Institutional Review Board of Asan Medical Center (IRB No. 2020-0518).

### 4.2. H. pylori Culture

Gastric tissue samples were collected from the antrum and corpus regions during esophagogastroduodenoscopy and immediately transferred to sterile Eppendorf tubes, which were placed in a vacuum container with dry ice for transportation. Upon arrival at the laboratory, specimens were stored in a deep freezer at –80 °C until processing. Prior to culture, the tissue samples were equilibrated to room temperature. For bacterial isolation, the specimens were cultured on specialized HP-selective medium consisting of *Brucella* broth agar enriched with 5% sheep blood and supplemented with antimicrobial agents (vancomycin 10 µg/L, trimethoprim 5 µg/L, amphotericin B 5 µg/L, and polymyxin B 2.5 IU). The inoculated plates were maintained at 37 °C in a microaerophilic environment (5% O_2_, 10% CO_2_, and 85% N_2_) for 5–7 days. HP identification was based on colony morphology, positive Gram staining characteristics, and urease activity.

### 4.3. Genomic DNA Extraction

Harvested pellets were resuspended in 200 µL PBS. Genomic DNA (gDNA) was extracted using the DNeasy^®^ (Qiagen, Hilden, Germany) kit. The mixture was incubated at 56 °C for 10 min after adding 20 µL proteinase K and 200 µL Buffer AL. Then, 200 µL ethanol was added and mixed by vortexing. The mixture was transferred to a DNeasy Mini spin column placed in a 2 mL collection tube and centrifuged at 6000× *g* for 1 min. A new 2 mL collection tube was used to add 500 µL Buffer AW1, followed by centrifugation for 1 min at 6000× *g*. A second new 2 mL collection tube was used to add 500 µL Buffer AW2, with centrifugation for 3 min at 20,000× *g*. For gDNA elution, 50 µL Buffer AE was added to the center of the spin column membrane and incubated for 1 min at room temperature. Centrifugation was then performed for 1 min at 6000× *g*. The gDNA was stored at –20 °C until required for polymerase chain reaction (PCR) amplification.

### 4.4. PCR Amplification

The PCR mixture included sense and antisense primers (Table 5), *Taq* PCR PreMix (AccuPower^®^, Daejeon, Republic of Korea), template DNA, and distilled water. PCR was performed under the following conditions: 10 min at 95 °C, followed by 30 cycles of 30 s at 95 °C, 30 s at each annealing temperature, and 30 s at 75 °C. After PCR, the products were run on 1% agarose gels, and bands were developed using an ultraviolet transilluminator and photographed.

### 4.5. Statistical Analysis

Descriptive variables were summarized as the median and interquartile range (IQR) or the mean and standard deviation (SD). Differences in patient characteristics and HP virulence factors between the EBV+ and EBV− groups were compared using independent *t*-tests and chi-square tests. Logistic regression analyses were conducted to investigate factors associated with EBVaGC, and odds ratios (ORs) and corresponding confidence intervals (CIs) were calculated. *p-*values < 0.05 were considered statistically significant. All statistical analyses were performed using SPSS version 24 (IBM Corporation, Somers, NY, USA).

## 5. Conclusions

In conclusion, our study demonstrates that patients in Korea with gastric cancer predominantly harbor highly virulent HP strains positive for *cagA*, *iceA*1, and *vacA*. The *ureA* virulence factor was associated with EBVaGC; however, it did not significantly influence clinical outcomes. These findings contribute to our understanding of the complex interactions between infectious agents in gastric carcinogenesis and underscore the need for further investigation into the molecular mechanisms underlying the relationship between HP virulence factors and EBV infection in gastric cancer development.

## Figures and Tables

**Table 1:** Comparison of clinical characteristics between EBV+ and EBV− patients among HP-positive patients (n = 54).

	EBV+ (n = 28)	EBV− (n = 26)	*p*-Value
Age at diagnosis, years, mean ± SD	61.5 ± 9.4	60.4 ± 12.8	0.310
Male sex, n (%)	23 (82.1)	22 (84.6)	1.000
Serum IgG Ab titer (n = 51), mean ± SD	4.8 ± 2.1	5.4 ± 2.3	0.149
Eradication success rate	15/17 (88.2)	15/16 (93.8)	1.000
Tumor size, cm, mean ± SD	2.9 ± 2.4	2.7 ± 1.9	0.808
Tumor location, n (%)			0.034
Upper	8 (28.6)	2 (7.7)	
Middle	15 (53.6)	12 (46.2)	
Lower	5 (17.9)	12 (46.2)	
Tumor type, n (%)			0.604
EGC	27 (96.4)	24 (92.3)	
AGC	1 (3.6)	2 (7.7)	
Histology, n (%)			<0.001
WD/MD	6 (21.4)	16 (61.5)	
PD/SRC	7 (25.0)	10 (38.5)	
GCLS	15 (53.6)	0 (0.0)	
AJCC TNM stage, n (%)			0.736
I	27 (96.4)	24 (92.3)	
II	0 (0.0)	1 (3.8)	
III	1 (3.6)	1 (3.8)	
Lymphovascular invasion, n (%)	2 (7.1)	4 (15.4)	0.413
Perineural invasion, n (%)	1 (3.6)	1 (3.8)	1.000
Treatment method, n (%)			0.821
ESD	17 (60.7)	15 (57.7)	
Surgery	11 (39.3)	11 (42.3)	
Recurrence-free survival, mean ± SD, median (IQR)	36.4 ± 17.0, 31.5 (31.0–32.0)	30.0 ± 5.1, 31.0 (23.3–46.5)	0.075
Overall survival, mean ± SD, median (IQR)	37.8 ± 16.1, 32.0 (31.0–32.0)	32.6 ± 3.7, 32.5 (24.3–46.5)	0.113

AGC, advanced gastric cancer; AJCC, American Joint Committee on Cancer; EBV, Epstein–Barr virus; EGC, early gastric cancer; ESD, endoscopic submucosal dissection; GCLS, gastric carcinoma with lymphoid stroma; HP, *Helicobacter pylori*; IQR, interquartile range; MD, moderately differentiated; TNM, tumor, node, and metastasis; PD, poorly differentiated; SD, standard deviation; SRC, signet ring cell carcinoma; WD, well differentiated.

**Table 2:** Positivity of virulence factors in EBV+ and EBV− HP colonies (n = 96).

	EBV+ (n = 46)	EBV– (n = 50)	*p*-Value
*cagA*, n (%)	46 (100.0)	50 (100.0)	1.000
*dupA*, n (%)	4 (8.7)	9 (18.0)	0.183
*iceA*1, n (%)	36 (78.3)	48 (96.0)	0.009
*iceA*2, n (%)	10 (21.7)	0 (0.0)	<0.001
*JHP917*, n (%)	0 (0.0)	9 (18.0)	0.003
*JHP918*, n (%)	4 (8.7)	9 (18.0)	0.183
*oipA*, n (%)	7 (15.2)	3 (6.0)	0.187
*16srRNA*, n (%)	46 (100.0)	50 (100.0)	1.000
*ureA*, n (%)	10 (21.7)	2 (4.0)	0.009
*vacA* s1, n (%)	46 (100.0)	50 (100.0)	1.000
s1a, n (%)	2 (4.3)	11 (22.0)	0.012
s1b, n (%)	46 (100.0)	50 (100.0)	1.000
s1c, n (%)	42 (91.3)	39 (78.0)	0.073
*vacA* m1, n (%)	46 (100.0)	50 (100.0)	1.000

EBV, Epstein–Barr virus; HP, *Helicobacter pylori*.

**Table 3:** Logistic regression analysis of the factors associated with EBV+ gastric cancer.

	Univariate Analysis		Multivariate Analysis	
	OR (95% CI)	*p*-Value	OR (95% CI)	*p*-Value
Age	1.010 (0.962–1.061)	0.691		
Sex (male)	0.836 (0.198–3.526)	0.808		
*dupA*	0.434 (0.124–1.520)	0.192		
*iceA*1	0.150 (0.031–0.727)	0.018	0.163 (0.032–0.819)	0.028
*JHP918*	0.434 (0.124–1.520)	0.192		
*oipA*	2.812 (0.681–11.605)	0.153		
*ureA*	6.667 (1.375–32.317)	0.018	6.148 (1.221–30.958)	0.028
*vacA* s1a	0.161 (0.034–0.772)	0.022		
*vacA* s1c	2.962 (0.870–10.077)	0.082		

CI, confidence interval; EBV, Epstein–Barr virus; OR, odds ratio.

**Table 4:** Comparison of clinical characteristics according to presence of *ureA* in EBV+ HP colonies (n = 46).

	*ureA*+ (n = 10)	*ureA*− (n = 36)	*p*-Value
Age at diagnosis, years, mean ± SD	66.6 ± 6.8	60.8 ± 10.1	0.114
Male sex, n (%)	6 (60.0)	30 (83.3)	1.189
Eradication success rate	5/5 (100.0)	10/12 (83.3)	0.669
Tumor size, cm, mean ± SD	2.8 ± 1.6	3.0 ± 2.8	0.723
Tumor location, n (%)			1.000
Upper	2 (20.0)	10 (27.8)	
Middle	6 (60.0)	20 (55.6)	
Lower	2 (20.0)	6 (16.7)	
Tumor type, n (%)			1.000
EGC	10 (100.0)	34 (94.4)	
AGC	0 (0.0)	2 (5.6)	
Histology, n (%)			0.697
WD/MD	1 (10.0)	7 (19.4)	
PD/SRC	2 (20.0)	10 (27.8)	
GCLS	7 (70.0)	19 (52.8)	
AJCC TNM stage, n (%)			1.000
I	10 (100.0)	34 (94.4)	
II	0 (0.0)	0 (0.0)	
III	0 (0.0)	2 (5.6)	
Lymphovascular invasion, n (%)	0 (0.0)	4 (11.1)	0.562
Perineural invasion, n (%)	0 (0.0)	2 (5.6)	1.000
Treatment method, n (%)			0.717
ESD	7 (70.0)	21 (58.3)	
Surgery	3 (30.0)	15 (41.7)	
Recurrence-free survival, mean ± SD, median (IQR)	36.2 ± 12.4, 43.0 (28.0–45.0)	36.4 ± 19.2, 43.0 (28.0–45.0)	0.423
Overall survival, mean ± SD, median (IQR)	37.9 ± 8.7, 27.5 (23.0–49.5)	38.3 ± 18.3, 29.5 (23.3–49.5)	0.503

AGC, advanced gastric cancer; EBV, Epstein–Barr virus; EGC, early gastric cancer; ESD, endoscopic submucosal dissection; GCLS, gastric carcinoma with lymphoid stroma; HP, *Helicobacter pylori*; IQR, interquartile range; MD, moderately differentiated; PD, poorly differentiated; SD, standard deviation; SRC, signet ring cell carcinoma; WD, well differentiated.

**Table 5:** PCR primers used to amplify the target sequences.

Primer	Sequence (5′-3′)	Annealing Temperature	Size (bp) of PCR Product
cagA	GAT AAC AGG CAA GCT TTT GAG G	55	349
	CTG CAA AAG ATT GTT TGG CAG A		
vacA s1	ATG GAA ATA CAA CAA ACA CAC	55	259
	CTG CTT GAA TGC GCC AAA C		
vacA s1a	TCT YGC TTT AGT AGG AGC	55	212
	CTG CTT GAA TGC GCC AAA C		
vacA s1b	AGC GCC ATA CCC CAA GAG	55	187
	CTG CTT GAA TGC GCC AAA C		
vacA s1c	CTY GCT TTA GTR GGG YTA	55	213
	CTG CTT GAA TGC GCC AAA C		
VAG (vacA m1)	CAA TCT GTC CAA TCA AGC GAG	50	570
	GCG TCT AAA TAA TTC CAA GG		
VA3	GGT CAA AAT GCG GTC ATG G	55	
	CCA TTG GTA CCT GTA GAA AC		
VA4	GGA GCC CCA GGA AAC ATT G	55	
	CAT AAC TAG CGC CTT GCA C		
ureA	GCC AAT GGT AAA TTA GTT	50	
	CTC CTT AAT TGT TTT TAC		
16s rDNA	CTG GAG AGA CTA AGC CCT CC	55	
	AGG ATC AAG GTT TAA GGA TT		
iceA1	GTG TTT TTA ACC AAA GTA TC	50	247
	CTA TAC CCA STY TCT TTG CA		
iceA2	GTT GGG TAT ATC ACA ATT TAT	50	229 or 334
	TTR CCC TAT TTT CTA GTA GGT		
OipA	CAA GCG CTT AGA TAG GC	50	427
	AAG GCA TTT TCT GCT GAA		
JHP917	TGG TTT CTA CTG ACA GAG CGC	55	307
	AAC ACG CTG ACA GGA CAA TCT CCC		
JHP918	CCCT ATA TCG CTA ACG CGC TCG	55	276
	AAG CTG AAG CGT TTG TAA CG		
dupA	TAA GCG TGA TCA CTC TGG AT	55	350
	TGG AAC GCC GCA TTC TAT TA		

PCR, polymerase chain reaction.

## Data Availability

The data presented in this study are available on request from the corresponding author. The data are not publicly available due to privacy.

## Supplementary Materials

The following supporting information can be downloaded at: https://www.mdpi.com/article/10.3390/antibiotics14060580/s1. Table S1. Comparison of clinical characteristics according to presence of *iceA*1 in EBV+ HP colonies (n = 46). Table S2. Comparison of clinical characteristics according to presence of *iceA*2 in EBV+ HP colonies (n = 46).
