# Supplementary material for: Distribution and Clinical Impact of Helicobacter pylori Virulence Factors in Epstein–Barr-Virus-Associated Gastric Cancer
Source: Antibiotics (Basel). 2025 Jun 5;14(6):580. doi: 10.3390/antibiotics14060580 (PMC12189602; doi:10.3390/antibiotics14060580)
Supplement: Supplementary file 1 [file antibiotics-14-00580-s001.zip › antibiotics-3627409-supplementary.pdf]

Supplementary Materials

Supplementary Table S1. Comparison of clinical characteristics according to presence of *iceA1* in EBV+ HP colonies (n=46)

	iceA1+ (n=36)	*iceA1*− (n=10)	*p*-value
Age at diagnosis, years, mean±SD	61.0±10.4	65.8±6.0	1.000
Male sex, n (%)	26 (72.2)	10 (100.0)	0.089
Eradication success rate	12/14 (85.7)	3/3 (100.0)	1.000
Tumor size, cm, mean±SD	3.3±2.8	1.9±1.3	0.056
Tumor location, n (%)			0.006
Upper	12 (33.3)	0 (0.0)	
Middle	16 (44.4)	10 (100.0)	
Lower	8 (22.2)	0 (0.0)	
Tumor type, n (%)			1.000
EGC	34 (94.4)	10 (100.0)	
AGC	2 (5.6)	0 (0.0)	
Histology, n (%)			0.879
WD/MD	6 (16.7)	2 (20.0)	
PD/SRC	10 (27.8)	2 (20.0)	
GCLS	20 (55.6)	6 (60.0)	
AJCC TNM stage, n (%)			1.000
I	34 (94.4)	10 (100.0)	
II	0 (0.0)	0 (0.0)	
III	2 (5.6)	0 (0.0)	
Lymphovascular invasion, n (%)	4 (11.1)	0 (0.0)	0.562

Perineural invasion, n (%)	2 (5.6)	0 (0.0)	1.000
Treatment method, n (%)			0.460
ESD	23 (63.9)	8 (80.0)	
Surgery	13 (36.1)	2 (20.0)	
Recurrence-free survival, mean±SD, median (IQR)	36.6±18.9, 28.0 (23.3–46.5)	35.8±13.8, 31.0 (25.5–46.0)	0.927
Overall survival, mean±SD, median (IQR)	38.8±17.5, 32.5 (24.3–46.5)	35.8±13.8, 31.0 (25.5–46.0)	0.684

EBV, Epstein–Barr virus; HP, *Helicobacter pylori*; IQR, interquartile range; SD, standard deviation.

Supplementary Table S2. Comparison of clinical characteristics according to presence of *iceA2* in EBV+ HP colonies (n=46)

	iceA2+ (n=10)	*iceA2–* (n=36)	*p*-value
Age at diagnosis, years, mean±SD	65.8±6.0	61.0±10.4	1.000
Male sex, n (%)	10 (100.0)	26 (72.2)	0.089
Eradication success rate	3/3 (100.0)	12/14 (85.7)	1.000
Tumor size, cm, mean±SD	1.9±1.3	3.3±2.8	0.056
Tumor location, n (%)			0.006
Upper	0 (0.0)	12 (33.3)	
Middle	10 (100.0)	16 (44.4)	
Lower	0 (0.0)	8 (22.2)	
Tumor type, n (%)			1.000
EGC	10 (100.0)	34 (94.4)	
AGC	0 (0.0)	2 (5.6)	
Histology, n (%)			0.879

WD/MD	2 (20.0)	6 (16.7)	
PD/SRC	2 (20.0)	10 (27.8)	
GCLS	6 (60.0)	20 (55.6)	
AJCC TNM stage, n (%)			1.000
I	10 (100.0)	34 (94.4)	
II	0 (0.0)	0 (0.0)	
III	0 (0.0)	2 (5.6)	
Lymphovascular invasion, n (%)	0 (0.0)	4 (11.1)	0.562
Perineural invasion, n (%)	0 (0.0)	2 (5.6)	1.000
Treatment method, n (%)			0.460
ESD	8 (80.0)	23 (63.9)	
Surgery	2 (20.0)	13 (36.1)	
Recurrence-free survival, mean±SD, median (IQR)	35.8±13.8, 31.0 (25.5–46.0)	36.6±18.9, 28.0 (23.3–46.5)	0.927
Overall survival, mean±SD, median (IQR)	35.8±13.8, 31.0 (25.5–46.0)	38.8±17.5, 32.5 (24.3–46.5)	0.684

EBV, Epstein–Barr virus; HP, *Helicobacter pylori*; IQR, interquartile range; SD, standard deviation.
